# Supplementary material for: Gut Microbiota Induced by Pterostilbene and Resveratrol in High-Fat-High-Fructose Fed Rats: Putative Role in Steatohepatitis Onset
Source: Nutrients. 2021 May 20;13(5):1738. doi: 10.3390/nu13051738 (PMC8160898; doi:10.3390/nu13051738)
Supplement: Supplementary file 1 [file nutrients-13-01738-s001.zip › nutrients-1211805-supplementary.pdf]

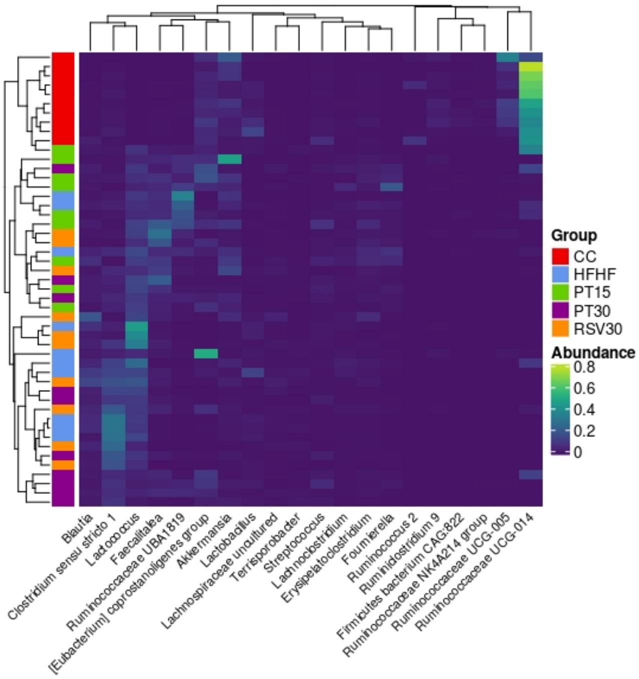

Figure S1. Heatmap of the hierarchical clustering of the samples based on the relative abundance of the 20 selected genera after Kruskal Wallis analysis showing an abundance greater than 0.01%. Bacterial genera are listed above, samples according to treatment groups are listed on the right. The intensity of the colour of the heatmap is directly proportional to the relative abundance of the genera in each sample.

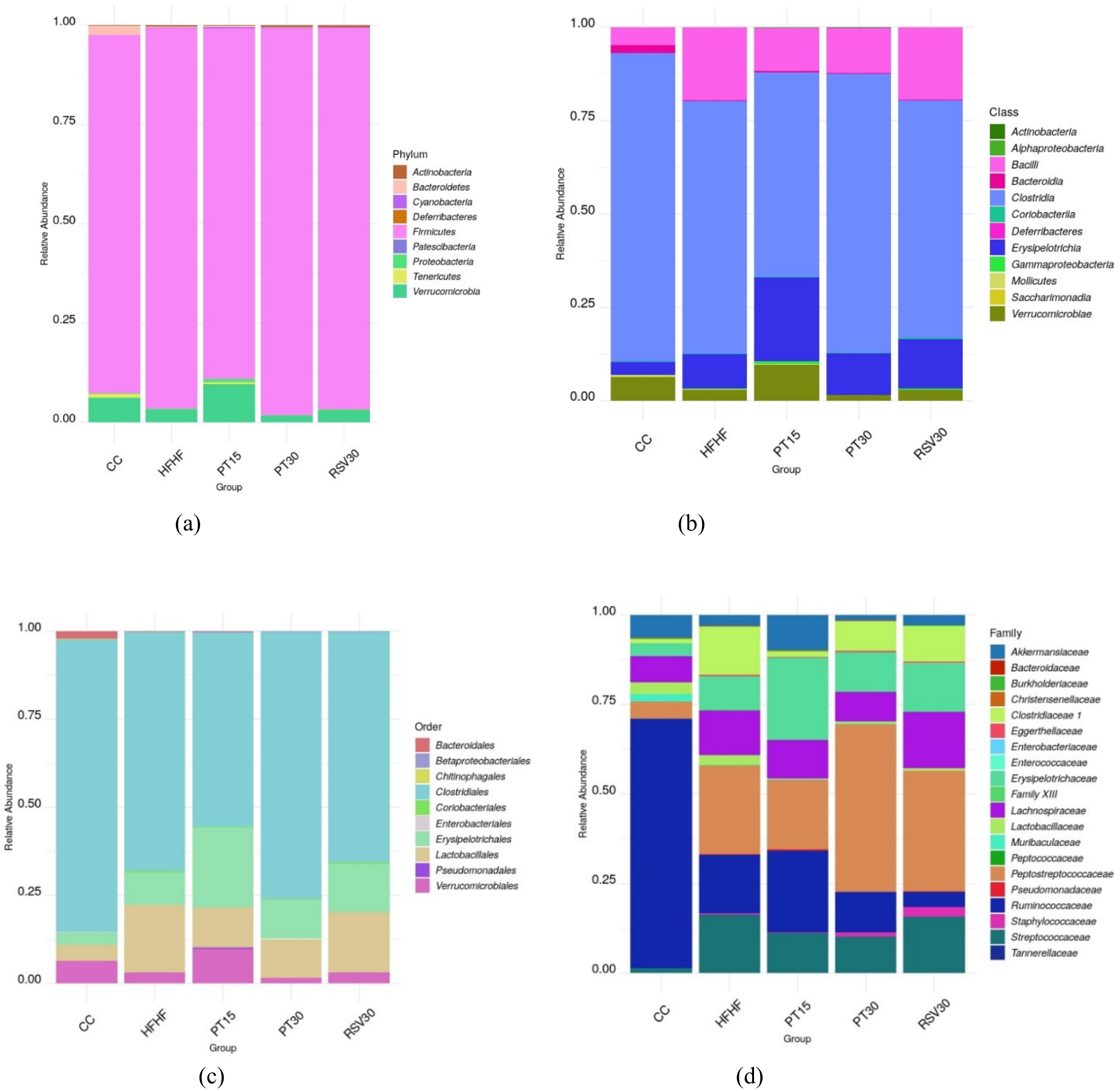

Figure S2. Relative abundances of (a) phylum, (b) class, (c) order and (d) family according to the different treatments.

Table S1: Spearmen Correlation values for twenty discriminatory microbes related with liver damage.

Spearman correlation (r) values for 20 discriminatory Genus related with liver damage characterised by liver steatosis and inflammation parameters, and elevated transaminase levels

Variable 1	Variable 2	Correlation (r)	p.adj (FDR)
Blautia	ALT	0.456905175597148	0.0180360723239981
Fournierella	ALT	0.416353941957414	0.0389835629570789
Lactococcus	ALT	0.52120191789825	0.00492451746094747
Terrisporobacter	ALT	0.611175077303858	8.66644929389796e-4
Blautia	AST	0.485063737451547	0.0100078883391401

Lactococcus	AST	0.556824073996308	0.00303483835150668
Blautia	Ballooning	0.506701084120134	6.50045561060904e-4
Clostridium sensu stricto 1	Ballooning	0.399274404867425	0.00815074074166622
Fournierella	Ballooning	0.32678422434165	0.0324738190945253
Lachnoclostridium	Ballooning	0.42845489633695	0.00429358989764311
Lactococcus	Ballooning	0.641968602991658	3.34976633708161e-5
Terrisporobacter	Ballooning	0.588020222656075	8.37668895258329e-5
Fournierella	Liver Weight	0.426385732285916	0.0370155680478647
Lactococcus	Liver Weight	0.453863681371537	0.020913493492184
Terrisporobacter	Liver Weight	0.521975844971278	0.00496338730413576
Blautia	Lobular inflammation	0.336973541655533	0.0270227000645993
Lactococcus	Lobular inflammation	0.506822768267648	6.50045561060904e-4
Terrisporobacter	Lobular inflammation	0.541055036004834	2.81028543074804e-4
Blautia	NAS score	0.409026553265179	0.0068714937576886
Clostridium sensu stricto 1	NAS score	0.354496977848419	0.0199176477787257
Lachnoclostridium	NAS score	0.349225752077074	0.0218271863553631
Lactococcus	NAS score	0.58423332832943	8.37668895258329e-5
Terrisporobacter	NAS score	0.549779348383312	2.28394345757734e-4
Blautia	Steatosis	0.386775022144831	0.0103938821319651
Faecalitalea	Steatosis	0.403629068521024	0.00749476364086627
Fournierella	Steatosis	0.445357211226763	0.00294376771826156
Lachnoclostridium	Steatosis	0.484333232553877	0.00120422475551721
Lactococcus	Steatosis	0.636035422574594	3.34976633708161e-5
Terrisporobacter	Steatosis	0.491349671076064	9.98618417613977e-4

Table S2: Spearmen Correlation values for twenty discriminatory microbes related with liver protection.

Spearman correlation (r) values for 20 discriminatory Genus with hepatic protective effect characterised by lower liver steatosis and inflammation parameters, and lower transaminase levels

Variable 1	Variable 2	Correlation (r)	p.adj (FDR)
Firmicutes bacterium CAG:822	ALT	-0.417339416581321	0.0389835629570789
Ruminiclostridium 9	ALT	-0.5916941817391	0.00105754588294675
Ruminococcaceae NK4A214 group	ALT	-0.521656161342467	0.00492451746094747
Ruminococcaceae UCG-005	ALT	-0.468785894945028	0.0136054216330726
Ruminococcus 2	ALT	-0.48840473847162	0.0100078883391401
[Eubacterium] coprostanoligenes group	Ballooning	-0.428173598793031	0.00429358989764311
Akkermansia	Ballooning	-0.383277082920363	0.0109348909855553
Firmicutes bacterium CAG:822	Ballooning	-0.554182351850268	2.06168282444313e-4
Ruminiclostridium 9	Ballooning	-0.613663089646971	4.3998502874691e-5
Ruminococcaceae NK4A214 group	Ballooning	-0.547989579216341	2.2940995554084e-4
Ruminococcaceae UCG-005	Ballooning	-0.584852345526097	8.37668895258329e-5
Ruminococcaceae UCG-014	Ballooning	-0.496008208867376	8.91575109677521e-4
Ruminococcus 2	Ballooning	-0.459917678016176	0.00228391154431472
Firmicutes bacterium CAG:822	Liver weight	-0.474331420798181	0.0136054216330726
Ruminiclostridium 9	Liver weight	-0.514532107152007	0.00569455907519946
Ruminococcaceae NK4A214 group	Liver weight	-0.54998725009252	0.00359570396687811
Ruminococcaceae UCG-005	Liver weight	-0.488379111010587	0.0100078883391401
Ruminococcus 2	Liver weight	-0.542801811635185	0.00376485846649832
[Eubacterium] coprostanoligenes group	Lobular inflamation	-0.562312372531265	1.63452882657477e-4
Akkermansia	Lobular inflamation	-0.444866306822603	0.00294376771826156
Firmicutes bacterium CAG:822	Lobular inflamation	-0.563387000915688	1.63452882657477e-4
Ruminiclostridium 9	Lobular inflamation	-0.623866430848481	3.34976633708161e-5
Ruminococcaceae NK4A214 group	Lobular inflamation	-0.625525016600599	3.34976633708161e-5
Ruminococcaceae UCG-005	Lobular inflamation	-0.602408820445455	6.21092576291637e-5
Ruminococcaceae UCG-014	Lobular inflamation	-0.447076157178561	0.00294376771826156
Ruminococcus 2	Lobular inflamation	-0.509401945836366	6.44739593936389e-4
Streptococcus	Lobular inflamation	-0.520810793917342	4.56212709823645e-4
[Eubacterium] coprostanoligenes group	NAS score	-0.454008477379222	0.00262632218245361
Akkermansia	NAS score	-0.388604026601062	0.0102955256891605
Firmicutes bacterium CAG:822	NAS score	-0.479757323766302	0.001341786668558
Ruminiclostridium 9	NAS score	-0.589217851844443	8.37668895258329e-5
Ruminococcaceae NK4A214 group	NAS score	-0.531065291934024	3.84124959355149e-4
Ruminococcaceae UCG-005	NAS score	-0.561899654044453	1.63452882657477e-4
Ruminococcaceae UCG-014	NAS score	-0.407314770973406	0.00700175669740708
Ruminococcus 2	NAS score	-0.44579687036522	0.00294376771826156
Streptococcus	NAS score	-0.38635231212271	0.0103938821319651
[Eubacterium] coprostanoligenes group	Steatosis	-0.368765603329902	0.0148985375369191
Akkermansia	Steatosis	-0.341572208304602	0.0250846086243554
Firmicutes bacterium CAG:822	Steatosis	-0.446825387794474	0.00294376771826156
Ruminiclostridium 9	Steatosis	-0.520667118388604	4.56212709823645e-4
Ruminococcaceae NK4A214 group	Steatosis	-0.528226786711421	4.0336297394543e-4
Ruminococcaceae UCG-005	Steatosis	-0.521519098403502	4.56212709823645e-4

Ruminococcaceae UCG-014	Steatosis	-0.440269540647754	0.00325671440712877
Ruminococcus 2	Steatosis	-0.461421862154589	0.00225815307369592
